# Inhalation of diesel exhaust does not exacerbate cardiac hypertrophy or heart failure in two mouse models of cardiac hypertrophy

**DOI:** 10.1186/1743-8977-10-49

**Published:** 2013-10-05

**Authors:** Yonggang Liu, Wei-Ming Chien, Ivan O Medvedev, Chad S Weldy, Daniel L Luchtel, Michael E Rosenfeld, Michael T Chin

**Affiliations:** 1 Division of Cardiology, Department of Medicine, University of Washington, Seattle, WA, USA; 2 Department of Environmental and Occupational Health Sciences, University of Washington, Seattle, WA, USA; 3 Center for Cardiovascular Biology, University of Washington School of Medicine, 850 Republican Street, Room 353, Box 358050, Seattle 98109, WA, USA

**Keywords:** Diesel exhaust, PM_2.5_, Air pollution, Cardiac hypertrophy, Heart failure

## Abstract

**Background:**

Strong associations have been observed between exposure to fine ambient particulate matter (PM_2.5_) and adverse cardiovascular outcomes. In particular, exposure to traffic related PM_2.5_ has been associated with increases in left ventricular hypertrophy, a strong risk factor for cardiovascular mortality. As much of traffic related PM_2.5_ is derived from diesel exhaust (DE), we investigated the effects of chronic DE exposure on cardiac hypertrophy and heart failure in the adult mouse by exposing mice to DE combined with either of two mouse models of cardiac hypertrophy: angiotensin II infusion or pressure overload induced by transverse aortic banding.

**Methods:**

Wild type male C57BL/6 J mice were either infused with angiotensin II (800 ng/kg/min) via osmotic minipump implanted subcutaneously for 1 month, or underwent transverse aortic banding (27 gauge needle 1 week for observing acute reactions, 26 gauge needle 3 months or 6 months for observing chronic reactions). Vehicle (saline) infusion or sham surgery was used as a control. Shortly after surgery, mice were transferred to our exposure facility and randomly assigned to either diesel exhaust (300 or 400 μg/m^3^) or filtered air exposures. After reaching the end of designated time points, echocardiography was performed to measure heart structure and function. Gravimetric analysis was used to measure the ventricular weight to body weight ratio. We also measured heart rate by telemetry using implanted ambulatory ECG monitors.

**Results:**

Both angiotensin II and transverse aortic banding promoted cardiac hypertrophy compared to vehicle or sham controls. Transverse aortic banding for six months also promoted heart failure in addition to cardiac hypertrophy. In all cases, DE failed to exacerbate the development of hypertrophy or heart failure when compared to filtered air controls. Prolonged DE exposure also led to a decrease in average heart rate.

**Conclusions:**

Up to 6-months of DE exposure had no effect on cardiac hypertrophy and heart function induced by angiotensin II stimulation or pressure overload in adult C57BL/6 J mice. This study highlights the potential importance of particle constituents of ambient PM_2.5_ to elicit cardiotoxic effects. Further investigations on particle constituents and cardiotoxicity are warranted.

## Background

There is extensive evidence from epidemiological investigations that acute and chronic exposure to fine ambient particulate matter (PM_2.5_) is associated with an increased risk of cardiovascular morbidity and mortality [1-5]. The biological mechanisms of PM_2.5_-mediated cardiotoxicity are still under active investigation, but recent studies have highlighted the potential for PM_2.5_ inhalation to impact cardiac hypertrophy and contractility [6-9]. In many urban regions, PM_2.5_ is largely derived from diesel exhaust (DE) [10], thus controlled exposure systems utilizing DE have been routinely used to investigate the cardiovascular effects of PM_2.5_ inhalation in both humans [11-14] and animals [15-20]. These studies have provided evidence that inhalation of DE can increase blood pressure [14], increase left ventricular ischemia [12], decrease vascular nitric oxide bioavailability [18,19], cause vasoconstriction [13], and increase sensitivity to vasoconstricting agents [18]. Although these systemic effects are believed to contribute to vascular disease, there is mounting evidence that these effects can result in cardiac hypertrophy in human populations.

Investigations by Van Hee and colleagues [7,8] have demonstrated that living in close proximity to a major roadway results in an increased exposure to PM_2.5_ and is associated with an increase in left ventricular mass. Left ventricular hypertrophy is an important predictor of cardiovascular mortality and can be a maladaptive response to increased blood pressure and left ventricular ischemia. In support of the role of particulate air pollution in causing this hypertrophic effect, evidence from Ying and colleagues [6] have shown that a 3-month exposure to concentrated ambient particulate matter (CAPs) in mice causes an exacerbation of a subsequent angiotensin II-induced cardiac hypertrophy in a Rho kinase dependent manner. Additionally, Wold and colleagues [9] have reported that a 9-month exposure to CAPs results in increased ventricular size along with systolic and diastolic dysfunction. In each of these studies, CAPs were collected from the Columbus, Ohio region, resulting in a particulate exposure that is a complex mixture from many sources. Currently, there are no studies to date investigating the acute and chronic effects of DE exposure alone on cardiac hypertrophy and failure. Here, we hypothesized that acute (1 week) and chronic (1, 3 and 6 months) exposure to DE (300 or 400 μg/m^3^, 6 hr/day, 5 days/week) would result in an exacerbation of cardiac hypertrophy and failure in mice when exposure occurred simultaneously with angiotensin II (Ang II) infusion or transverse aortic constriction (TAC).

In the work presented here, we report that DE exposure failed to have any significant effect on susceptibility to cardiac hypertrophy or failure in either the Ang II infusion or TAC models of heart failure following a 1-week, 1-month, 3-month, or 6-month exposure. These observations lead us to conclude that the observed effects on PM_2.5_-induced hypertrophy may be a unique response to the complex exposure in CAPs, whereas DE alone is insufficient. These data highlight the need for continued investigation into multi-pollutant models of air pollution and their potential effects on cardiac hypertrophy and failure.

## Results

### Effects of diesel exhaust exposure combined with angiotensin II infusion

**Figure 1:**
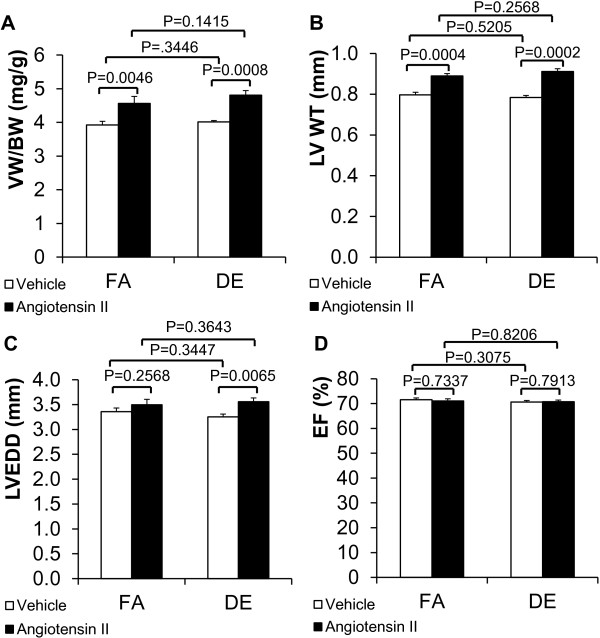
**Effects of diesel exhaust exposure and angiotensin II on cardiac hypertrophy.** Angiotensin II was infused (800 ng/kg/min) via osmotic minipump in conjunction with either DE (300 μg/m^3^, non-ozone treated) or FA exposure for 4 weeks (6 hr/day, 5 days/week). **A**. Ventricular weight to body weight ratio (VW/BW). N = 8 for all groups. **B**. Left ventricular wall thickness (LV WT). N = 10 for all groups. **C**. Left ventricular end diastolic dimension (LVEDD). N = 10 for all groups. **D**. Left ventricular ejection fraction (LVEF). N = 10 for all groups. FA: filtered air. DE: diesel exhaust.

To test the effect of DE exposure on cardiac hypertrophy induced by Ang II, we implanted osmotic minipumps for the infusion of Ang II or saline vehicle and then exposed mice to either DE or filtered air (FA). Echocardiography measurements and gravimetric analysis were performed after 1 month. As expected, there were increases in left ventricular wall thickness (LV WT) and ventricular weight to body weight ratio (VW/BW) in Ang II infusion groups compared with vehicle groups with both DE and FA exposures (Figure 1A and B), but there were no differences between DE exposed mice and FA controls (Figure 1A and B). Left ventricular ejection fraction (LVEF) was not affected by Ang II or DE (Figure 1D). Left ventricle end diastolic dimension (LVEDD) was mildly increased by angiotensin II in the DE exposed animals compared to vehicle controls but there was no effect of angiotensin II exposure in FA exposed animals (Figure 1C).

### Effects of short-term diesel exhaust exposure combined with acute pressure overload

**Figure 2:**
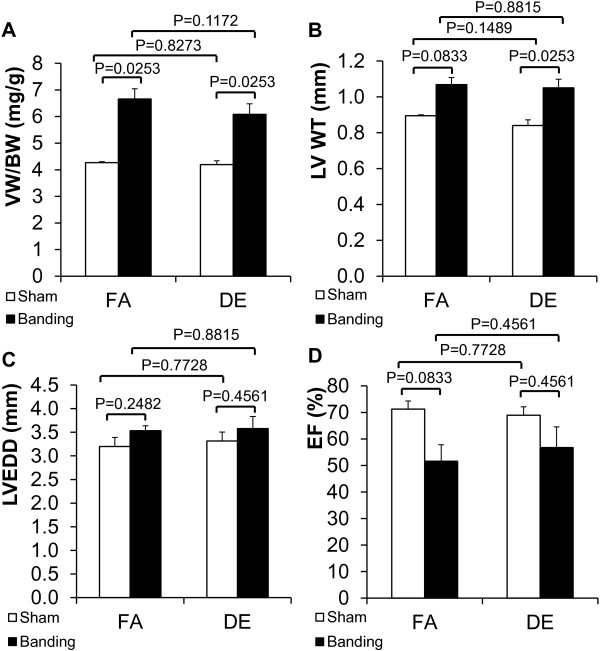
**Effects of short-term diesel exhaust exposure and acute pressure overload on cardiac hypertrophy.** Transverse aortic banding (27 gauge needle) was done in conjunction with DE (400 μg/m^3^, ozone treated) or FA exposure for one week (6 hr/day, 5 days/week). **A**. Ventricular weight to body weight ratio (VW/BW). FA/sham: n = 3; FA/banding: n = 5; DE/sham: n = 3; DE/banding: n = 5. **B**. Left ventricular wall thickness (LV WT). FA/sham: n = 2; FA/banding: n = 3; DE/sham: n = 3; DE/banding: n = 5. **C**. Left ventricular end diastolic dimension (LVEDD). FA/sham: n = 2; FA/banding: n = 3; DE/sham: n = 3; DE/banding: n = 5. **D**. Left ventricular ejection fraction (LVEF). FA/sham: n = 2; FA/banding: n = 3; DE/sham: n = 3; DE/banding: n = 5. FA: filtered air. DE: diesel exhaust.

We performed transverse aortic banding on wild type C57BL/6 J mice with a 27 g needle to induce cardiac hypertrophy and exposed the mice to 400 μg/m^3^ ozone treated DE or FA for 1 week. After the procedures, VW/BW and LV wall thickness trended towards an increase in transverse aortic banding groups compared with sham groups but there was no difference between DE and FA exposure groups (Figure 2A,B). Measurement of heart function and LV chamber size after transverse aortic banding suggested a trend towards an increase in LVEDD and a decrease in LVEF when compared to sham operated controls but these changes did not reach statistical significance. We also observed no trend or significant differences in LVEDD or LVEF between DE and FA exposure groups (Figure 2C,D). Our sample size is small, however, and we cannot absolutely rule out type II error. To address this issue we performed a sample size calculation to determine what sample size would be needed to achieve sufficient statistical power to determine whether the slight difference between the DE TAC and FA TAC groups is significant. This calculated sample size is 37 mice, which is impractical, and suggests that any significant effect of diesel exhaust would likely be exceedingly small if present at all.

### Effects of long-term diesel exhaust exposure combined with chronic pressure overload

**Figure 3:**
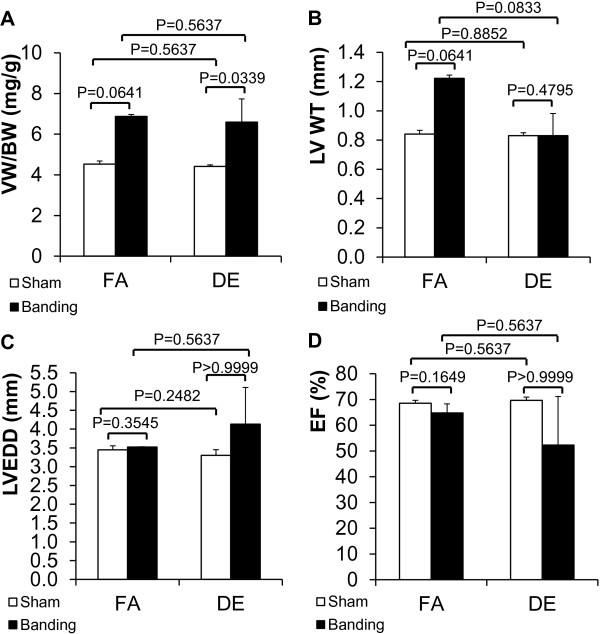
**Effects of 3 months of diesel exhaust exposure and chronic pressure overload on cardiac hypertrophy.** Transverse aortic banding (26 gauge needle) was done in conjunction with DE (300 μg/m^3^, non-ozone treated) or FA exposure for 3 months (6 hr/day, 5 days/week). **A**. Ventricular weight to body weight ratio (VW/BW). **B**. Left ventricular wall thickness (LV WT). **C**. Left ventricular end diastolic dimension (LVEDD). **D**. Left ventricular ejection fraction (LVEF). FA/sham: n = 4; FA/banding: n = 2; DE/sham: n = 4; DE/banding: n = 3. FA: filtered air. DE: diesel exhaust.

Since we failed to observe a difference in acute exposure experiments, we investigated the effects of prolonged diesel exposure on TAC induced cardiac hypertrophy. We used a 26 g needle for the transverse aortic banding to allow survival of the mice for the duration of the DE exposure. After surgery, mice were exposed to either 300 μg/m^3^ non-ozone treated DE or FA for 3 months. At the end of the study period, VW/BW showed a trend towards an increase in transverse aortic banding groups compared with sham operated groups but there was no difference between DE and FA groups (Figure 3A). Interestingly, there was a trend towards increased LV wall thickness after banding in the FA controls that was not seen in the DE exposed animals (Figure 3B), while there was a trend towards increased LV chamber size after TAC in the DE exposed animals that was not seen in the FA controls (Figure 3C). There was also a trend towards a decrease in LVEF in the TAC groups compared to sham operated groups, and also a trend towards decreased LVEF in the DE TAC group compared to the FA TAC and FA sham groups, but these trends were not statistically significant (Figure 3D). Our sample size is small, so we performed sample size calculations to determine the number of animals needed to demonstrate significant differences if these effects were real, and determined that we would need a group size of 68 for LVEDD and 60 for LVEF to see a difference between FA TAC and DE TAC. Although we cannot absolutely rule out type II error, these findings again indicate that any significant effect of diesel exhaust would likely be exceedingly small if present at all.

**Figure 4:**
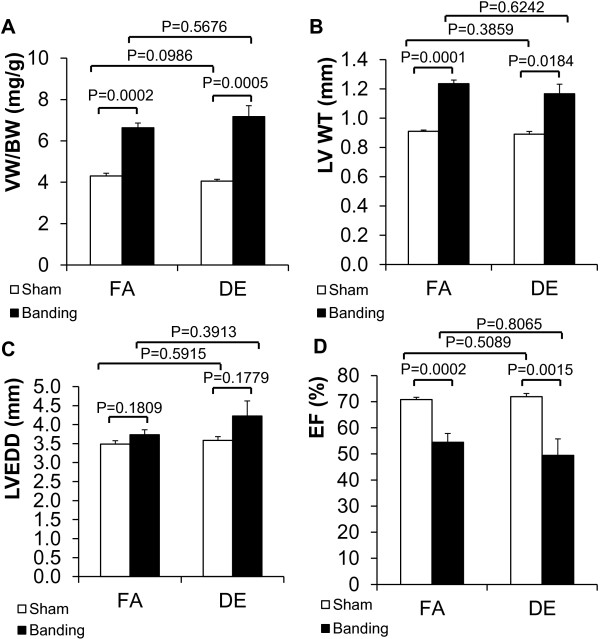
**Effects of 6 months of diesel exhaust exposure and chronic pressure overload on cardiac hypertrophy.** Transverse aortic banding (26 gauge needle) was done in conjunction with DE (300 μg/m^3^, non-ozone treated) or FA exposure for 6 months (6 hr/day, 5 days/week). **A**. Ventricular weight to body weight ratio (VW/BW). **B**. Left ventricular wall thickness (LV WT). **C**. Left ventricular end diastolic dimension (LVEDD). **D**. Left ventricular ejection fraction (LVEF). FA/Sham: n = 11; FA/Banding: n = 10; DE/Sham: n = 8; DE/Banding: n = 9. FA: filtered air. DE: diesel exhaust.

To determine whether a more prolonged exposure to diesel exhaust would demonstrate significant effects on hypertrophy and ventricular function, we extended the exposure time to 6 months and repeated the study. Again, we performed 26 g transverse aortic banding on wild type C57BL/6 J mice to induce cardiac hypertrophy and exposed the mice to 300 μg/m^3^ non-ozone treated DE or FA. After the procedures, VW/BW increased significantly in TAC groups compared with sham groups, but there was no effect of DE compared to FA (Figure 4A). LV WT increased after TAC, however, there was no difference between DE and FA exposure (Figure 4B). LVEDD showed a trend towards enlargement in the TAC groups with a trend towards exacerbation by DE exposure, but the differences were not statistically significant (Figure 4C). LVEF decreased significantly after aortic banding in both DE and FA groups (Figure 4D), but there were no significant differences between DE and FA exposure groups.

### Effects of long-term diesel exhaust exposure on heart rate

**Figure 5:**
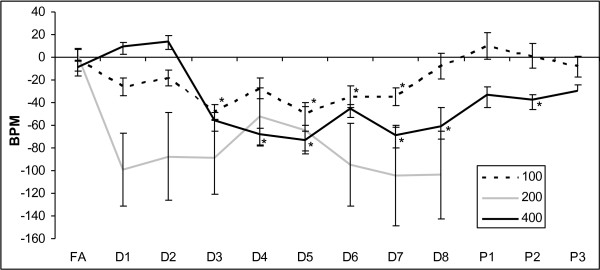
**Effects of diesel exhaust exposure on heart rate.** Change from baseline heart rate levels in beats per minute (BPM) during exposure to FA followed by 100, 200, or 400 μg/m^3^ of DE for eight weeks (D1 – D8) and in the three weeks following that where there was no exposure (P1 – P3). No data was collected for the group exposed to 200 μg/m^3^ after D8. Data represent the mean +/− SEM. * p-value < 0.05 compared to FA.

As expected, there were no changes in HR during the week of FA for any of the groups. With DE exposure, there was a significant decrease in HR that persisted until the DE exposure concluded. There was slight recovery of HR in the post-exposure weeks (Figure 5). Interestingly, there was no dose–response relationship noted. A similar decrease was noted following exposure to all three doses of DE, with no significant difference between the three doses. There was greater apparent variability noted in the DE_200_ group, but this is due to the smaller study group and not actual increased variability.

## Discussion

In this study, we investigated the hypothesis that the inhalation of DE under both acute (1-week, 1-month) and chronic (3-month, 6-month) conditions would exacerbate the progression of cardiac hypertrophy and failure in two models of cardiac hypertrophy in mice. Although there is strong evidence that exposure to traffic related air pollution results in increased left ventricular mass in humans [7,8], and results in increased hypertrophy as well as systolic and diastolic dysfunction in mice [9], we did not observe any effect of DE exposure alone on the hypertrophic or contractile responses in two mouse models of cardiac hypertrophy.

We did observe a paradoxical effect of DE exposure on heart rate. In epidemiological studies, the general consensus is that HR increases while measures of HRV decrease in humans exposed to PM [4]. However, we see the opposite effect in this study. The reason for this may be due to differences between rodents and humans. Other studies have seen similar results with exposure to CAPs [21] and carbon black [22]. It is possible that although the autonomic nervous system (ANS) is affected by the DE exposure, afferent ANS pathways differ in mice from those in humans.

There are some caveats to our study. Although we do not observe any effects of DE exposure on hypertrophy or heart function at the organ level, it is possible that activation of pathways at the molecular level has occurred. Even if these changes are occurring, however, the lack of response at the organ level indicates that these hypertrophy models and this exposure system may be limited in their utility for studying cardiovascular pathogenetic mechanisms induced by DE exposure. Another caveat is that our echocardiographic assessment was done from 1–7 days after diesel exposure was terminated, and we cannot rule out short lived effects on cardiac function that disappear rapidly after exposure is terminated. Others have found, however, that CAP exposure can lead to significant effects on cardiac function two weeks after the exposure is terminated [6]. Using the same exposure facility and conditions described here, we have also found that exposure to DE exhaust in utero and early in life can predispose to hypertrophy in adults, long after the exposure is terminated (Weldy CS et al., manuscript submitted). Another caveat to our study is that we did not measure blood pressure in the animals that were treated with Angiotensin II, and there is a possibility that the minipumps failed to deliver appropriate amounts. Our observation of Ang II induced increases in VW/BW and LV WT in Figure 1 is strongly indicative that Ang II was infused appropriately. Angiotensin II is also known to promote cardiac hypertrophy at subpressor doses, obviating the need for blood pressure monitoring [23]. Still another potential caveat is that we did not assess for either pulmonary or systemic inflammation in our mice. We and others have shown that similar exposures using our facility result in low level neutrophilic airway inflammation [24] and systemic lipid peroxidation [25]. We expect similar results in the mice used in this study.

Controlled exposures to DE in both human and animal studies have been used routinely to model ambient PM_2.5_ exposure. The benefits of using DE have been the relative ease of reproducible and well-defined exposures as well as the ability to compare biological effects of DE across institutions. In addition, in many regions, the majority of ambient PM_2.5_ is derived from diesel engines, thus DE exposures are largely mimicking ambient exposures to urban and traffic related particulate air pollution. Studies utilizing collected diesel exhaust particulate (DEP) in both in vitro and in vivo exposures have demonstrated their robust proinflammatory effects and their strong potential to influence systemic vascular function [26-29], but there is strong evidence to suggest that the inflammatory and toxic potential of these particles is influenced by additional particulate constituents, such as endotoxin and metals.

The disadvantage of using DE is that it is a relatively ‘simple’ exposure model, in that the PM_2.5_ resulting from DE is mostly carbonaceous in nature, containing polycyclic aromatic hydrocarbons and redox active quinones, but lacking many other contaminants important to particulate matter toxicity. In the work reported in Ying et al. [6] and Wold et al. [9], they report their observed effects on cardiac hypertrophy and failure after exposure to CAPs from the Columbus, Ohio region. In both of these studies, exposures were done using ambient particulate material that was concentrated directly from the atmosphere, resulting in an exposure that consists of PM_2.5_ from traffic related sources such as diesel exhaust, but also from jet fuel (as an airport is near the exposure site), agriculture (which may result in endotoxin exposure), as well as coal fired power plants and other industrial sources of PM_2.5_. The advantage of these CAPs studies is that the PM_2.5_ exposures closely match the human exposures in the population. The main disadvantage is that these exposures are difficult to compare across institutions as PM_2.5_ composition will vary widely across regions and proximity to sources, and the PM_2.5_ composition during the exposure may vary from day-to-day depending on the magnitude of PM_2.5_ emissions near the exposure site.

The results from the studies reported here suggest that a DE exposure in C57BL/6 J mice up to 6 months in length has no effect on the cardiac hypertrophic or functional responses in Ang II infusion or TAC models of heart failure. That we do not observe any effect in these studies whereas others have reported effects using CAPs suggests that either 1) 6 months exposure to DE is not enough time to see any effect, or 2) DE exposures are lacking PM constituents found in CAPs that provide for their unique cardiotoxicity.

It is possible that a 6 month exposure to DE is not enough time to elicit the hypertrophic or functional effects on the mouse heart. In the report published by Wold et al. [9], their CAP exposure was for 9 months, and this exposure resulted in increased expression of hypertrophic markers, decreased fractional shortening, diastolic dysfunction and decreased contractile reserve, consistent with heart failure. In our exposures, diesel exhaust concentration is dynamically regulated to 300 μg/m^3^, 6 hrs a day, 5 days a week. Since mice are exposed to FA for 18 hrs a day, and during weekends, our total time weighted average exposure for the 6 month period is expected to be ~53 μg/m^3^/hr. We calculate cumulative exposure, using concentration x time, to be 53 μg/hr × 6 months × 30 days/month × 24 hrs/day = 228960 μg/m^3^. In the study by Wold et al. [9], they calculated a time weighted average exposure to be 15 μg/m^3^/hr. The calculated cumulative exposure over 9 months is 15 μg/m^3^ × 9 months × 30 days/month × 24 hrs/day = 97200 μg /m^3^. In our assessment, our cumulative exposure to PM_2.5_ in 6 months is 2.36 times greater than their exposure in 9 months. This suggests to us that our inability to find any significant effect on cardiac hypertrophy or function with our 6-month DE exposure must be due to either 1) constituents of CAPs within the Wold et al. study that elicit greater cardiotoxicity than PM_2.5_ from DE, or 2) duration of PM_2.5_ exposure is more important than cumulative exposure. Although, from this assessment, we cannot rule out the possibility that the duration of exposure is the critical component to observing cardiotoxicity, we believe this is unlikely, as our 2.36 times greater cumulative PM_2.5_ exposure would be expected to elicit a more rapid acceleration of cardiac hypertrophy and failure. In addition, although Wold et al. [9] had a longer exposure time than the studies reported here, Ying et al. [6] exposed mice to CAPs for only 3 months, and then assessed their susceptibility to Ang II-induced cardiac hypertrophy. They observed the 3-month CAPs exposure to promote cardiac remodeling induced by a two-week infusion of Ang II through a Rho kinase dependent mechanism. The report by Ying et al. [6] suggests that a 3-month exposure to CAPs is sufficient to observe these adverse effects resulting from Ang II infusion. In this study, their cumulative PM_2.5_ exposure was 15 μg/m^3^/hr × 3 months × 30 days/month × 24 hrs/day = 32400 μg/m^3^. Our study has a cumulative exposure that is 7 times greater than their study, further suggesting that our lack of finding a significant effect is likely due to diesel exhaust particulate lacking PM constituents that elicit unique responses to the myocardium.

There is a growing belief that particular constituents of ambient PM_2.5_ likely have important modifications to their toxicity within human populations. In a recent report by Wu et al. [30], acute PM_2.5_ exposures in a Chinese population were associated with increases in systolic as well as diastolic blood pressure, and these increases in BP were found to be positively modified by PM_2.5_ constituents such as carbonaceous fractions, ions, and metals. In addition, a recent report by Kim et al. [31] observed PM_2.5_ constituents such as sulfate and nitrate to alter their observed temporal relationships between increases in PM_2.5_ and hospital admissions. The potential for PM constituents to alter their cytotoxic and proinflammatory effects have been investigated *in vitro*, where the presence of metals such as arsenic, zinc, chromium, copper, manganese, and iron have been found to be positively associated with deleterious effects [32-35]. As the cytotoxic and proinflammatory effects of PM_2.5_ have been suggested to be largely mediated by the generation of reactive oxygen species and subsequent oxidative stress, it is likely the case that metal constituents that can participate in redox cycling and Fenton chemistry are capable of enhancing PM_2.5_ toxicity. As we do not observe a chronic DE exposure (which is largely carbonaceous, containing low metal content) to elicit changes in cardiac hypertrophy or function, it is possible that metal constituents within CAPs are likely enhancing PM_2.5_ effects on pulmonary and systemic inflammation, potentially driving these changes in ventricular remodeling. These observations may suggest that a certain ‘threshold’ of inflammation and oxidative stress is required to be exceeded before changes in cardiac size or function will be observed. In support of this hypothesis, it has been recently reported that rapid, high dose exposures of collected DEP to rats via intranasal nebulization over a 5-week period can elicit cardiac dysfunction and remodeling [35], suggesting that DEP may be able to elicit these effects, but it may require extremely high doses, rapid administration, and lengthy exposure times that exceed what would be required when particle constituents within CAPs are included.

## Conclusions

Here, we report that a DE exposure (300 μg/m^3^, 6 hr/day, 5 days/week for up to 6 months) has no effect on cardiac hypertrophy and function in two models of cardiac hypertrophy. These observations suggest that the specific effects that have been previously reported on PM_2.5_ on cardiac hypertrophy and heart failure may be due to unique PM constituents found in complex mixtures of ambient PM_2.5_, but not in DE. Ambient particulate air pollution is an important public health concern. In the most recent Global Burden of Disease report, ambient particulate air pollution is the #9 cause of disease worldwide [36]. Regulatory policies and guidelines will need to take into account the complexity of PM_2.5_ exposures across regions and will need to evaluate the importance of multi-pollutant models of air pollution. Further investigation is needed to understand the PM constituents that produce these cardiotoxic effects.

## Methods

### Animals

Wild type male C57BL/6 J mice (10 weeks old for angiotensin II infusion and 12 weeks old for transverse aortic banding surgery) were used for these studies. Mice were obtained from Jackson Laboratories (Bar Harbor, ME). The studies were conducted according to guidelines and protocols approved by the University of Washington Institutional Animal Care and Use Committee.

### Angiotensin II infusion

Angiotensin II (Ang II) or vehicle (saline) was infused continuously through a subcutaneously implanted osmotic minipump (Alzet 1004, DURECT Corporation, Cupertino, CA) as previously described [37,38]. The Ang II infusion rate was 800 ng/kg/min for 4 weeks. Shortly after surgery, mice were transferred to the diesel exposure facility, where they were exposed to either DE (300 μg/m^3^, non-ozone treated) or filtered air (FA) for 6 hours per day, 5 days per week for 4 weeks. After the exposure, mice were housed in room air for 1 week before echocardiographic assessment and gravimetric analysis.

### Transverse aortic banding

Mice underwent transverse aortic banding surgery or sham surgery as previously described [39-41]. For short term exposure experiments, transverse aortic banding was performed using a 27 gauge needle followed by either DE exposure (400 μg/m^3^, ozone treated), or FA exposure for 6 hours per day, 5 days per week, for 1 week. Echocardiography was performed 9 days after the surgery and gravimetric analysis was performed 10 days after the surgery.

For the chronic exposure experiments, transverse aortic banding was performed using a 26 gauge needle followed by either DE exposure (300 μg/m^3^, non-ozone treated) or FA exposure for 6 hours per day, 5 days per week for either 3 or 6 months. Echocardiography and gravimetric analyses were performed 3 or 6 months after the surgery.

### Diesel exhaust exposure

Following minipump implantation or TAC surgery, mice were transferred to our diesel exposure facility where exposures to either FA or DE were conducted simultaneously in an Allentown caging system (Allentown, NJ, USA) under SPF conditions. DE (300 or 400 μg/m^3^) was generated from a single cylinder Yanmar diesel engine (model YDG5500EV-6EI) operating on 75% load as previously described [20,42,43]. The stability of the PM_2.5_ concentration is maintained by an elaborate feedback control system [42]. Exposures were 6 hr/day, 5 days/week, producing roughly a 50 μg/m^3^/hr cumulative time weighted average exposure. A DE exposure concentration of 300 μg/m^3^ was chosen as this is a relevant exposure concentration in certain occupational settings such as mining [44], and is consistent with prior studies investigating the vascular effects of acute DE inhalation [15,17-19]. A dose of 400 μg/m^3^ was chosen in the short term TAC study to provide the maximum possible DE exposure. In that same study, we also treated the DE particles with ozone to mimic atmospheric conditions and enhance the toxicity of the particles. No ozone was introduced into the exposure chamber. In our system, we generally observe carbon monoxide concentrations of 2 ppm at 300 μg/m^3^ PM_2.5_. At this PM concentration, NO_2_ is generally 6 ppb, while total NO_x_ is approximately 1700 ppb. Most of the NO_x_ is in the form of NO, due to lack of exposure to sunlight in our system. We do not expect these gaseous components to affect heart failure progression. In general, our DE composition is very similar to the Environmental Protection Agency profile for light diesel exhaust, with the exception of the NO to NO_2_ ratio [42].

### Echocardiography

Echocardiographic experiments was performed to measure heart function and chamber dimensions such as left ventricular wall thickness (LV WT), left ventricular end diastolic dimension (LVEDD), and left ventricular ejection fraction (LVEF, in%) using a VisualSonics VEVO 770 system equipped with a 707B scan head, as previously described [39,40]. Mice were lightly anesthetized with 1% isoflurane when performing echocardiography. Data were accumulated in M mode from the short axis and LVEF (in%) was calculated as previously described [39,40].

### Gravimetric analysis

Gravimetric analysis was performed as previously described [39,40]. The mice were euthanized by carbon dioxide inhalation followed by weighing, heart removal and exsanguination. Atria were removed and ventricular weight changes between groups were tabulated as mg ventricular weight (VW) / g body weight (BW) and mg ventricular weight (VW) / mm tibia length (Tibia).

### Measurement of heart rate

Mice were implanted with radiotelemetric ECG devices (TA10ETA-F20) from Data Sciences International (Minneapolis, MN). The mice were anaesthetized with 2% isofluorane. A subcutaneous pocket was made by blunt dissection in the right flank and the transmitter inserted. The leads were placed in a Lead II position with the cathode at the right shoulder and the anode on the animal’s left side just below the rib cage. The transmitter and leads were sutured to secure placement and the mouse was allowed to recover for a minimum of one week prior to baseline data collection.

ECG, heart rate (HR) and animal activity count data were collected using Dataquest A.R.T. software from DataSciences, Incorporated (Minneapolis, MN). Data were collected for 30 seconds every 5 minutes. Heart rate was collected in beats per minute (BPM) and averaged over a 12 hour time frame based on whether the lights were on (rest) or off (wake) in the animal room.

### Statistical analyses

All data were reported as mean ± SEM. The comparison between the groups was made by a Mann–Whitney test with Bonferroni correction. Since we generally made 4 pairwise comparisons for each measurement, a value of p > 0.0125 was considered as no difference between any two groups. A value of p < 0.0125 was considered as significantly different between groups. All the analyses were performed using commercially available software (StatView, SAS Institute, Inc., Cary, NC).

Sample size calculation to achieve statistical power of 0.8 was performed through using online calculator provided by Professor Rollin Brant, Department of Statistics, University of British Columbia (http://www.stat.ubc.ca/~rollin/stats/ssize/n2.html) [45].

## Competing interests

The authors declare that they have no competing interests.

## Authors’ contributions

YL carried out the surgical procedures, arranged for diesel exposure, performed echocardiography, gravimetry, figure generation, statistical analysis and helped draft the manuscript. WMC assisted with the mouse husbandry, angiotensin II infusion and 6 month exposure experiments. IOM assisted with the statistical analysis. CSW helped draft and edit the manuscript. DLL and MER provided the heart rate data. MTC conceived of the study, participated in its design and coordination and helped to draft and edit the manuscript. All authors read and approved the final manuscript.

## Authors’ information

YL is a junior faculty member and cardiovascular scientist. WMC is a staff research scientist. IOM is a clinical cardiology fellow. CSW is a postdoctoral fellow and toxicologist. DLL is a senior faculty member and toxicologist. MER is a senior faculty member and pathologist. MTC is a senior faculty member, cardiovascular scientist and practicing cardiologist.
